# ECG changes following balloon pulmonary angioplasty in patients with chronic thromboembolic pulmonary hypertension: a retrospective study

**DOI:** 10.1186/s13019-024-02960-z

**Published:** 2024-08-21

**Authors:** Tao Guo, Xiao-mei Zeng, Hou-quan Huang, Xiao-feng Wu, Wen-liang Guo, Hai-ming Chen, Qiao-nan Zhong, Xin Yang, Hui-ling Ye, Cheng Hong

**Affiliations:** 1 Department of Cardiovascular Medicine, the First Affiliated Hospital of Guangzhou Medical University, Guangzhou, 510010 China; 2 Department of General Practice, Shenzhen Second People’s Hospital, The First Affiliated Hospital of Shenzhen University, Shenzhen, China; 3 The State Key Laboratory of Respiratory Disease, National Clinical Research Center for Respiratory Disease, Guangzhou Institute of Respiratory Health, the First Affiliated Hospital of Guangzhou Medical University, 151 Yanjiang Road, Guangzhou, 510010 China; 4 Department of General Practice, the First Affiliated Hospital of Guangzhou Medical University, Guangzhou, 510010 China; 5 The Department of electrocardiography, the First Affiliated Hospital of Guangzhou Medical University, Guangzhou, 510010 China

**Keywords:** Chronic thromboembolic disease pulmonary hypertension, Balloon pulmonary angioplasty, Electrocardiogram, Hemodynamics

## Abstract

**Purpose:**

This research evaluates the effect of balloon pulmonary angioplasty (BPA) on cardiac electrophysiological changes in patients with chronic thromboembolic pulmonary hypertension (CTEPH).

**Methods:**

Involving a retrospective analysis of 39 CTEPH patients (average age 61 ± 11), who had at least two BPAs and paired ECGs pre- and post-surgery, we examined changes in ECG indicators of right ventricular hypertrophy and their correlation with hemodynamic results.

**Results:**

BPA yielded marked improvements in cardiac function and hemodynamics. ECG parameters, specifically the Lewis criteria and Butler-Leggett score, correlated strongly with hemodynamics and were predictive of a mean pulmonary arterial pressure (mPAP) ≥ 35mmHg. Notably, QRS complex axis normalization was observed in 25 patients, with 14 fully normalizing (range − 30° to + 90°). The qR pattern in V1 vanished in 9 cases, and 75% of the patients in qR pattern in V_1_ group had QRS complex electrical axis completely returned to normal range. The qR V1 group had higher mPAP and pulmonary vascular resistance (PVR), and lower cardiac output and index compared to the non-qR V1 group, alongside a higher Butler-Leggett score.

**Conclusions:**

BPA enhances cardiac function and hemodynamics in CTEPH patients, with certain ECG measures such as Lewis criteria and Butler-Leggett score reflecting the severity of hemodynamic impairment. The reversal of QRS axis deviation and the disappearance of the qR pattern in lead V_1_ may serve as valuable indicators for assessing post-BPA satisfaction in CTEPH patients.

## Introduction

Chronic thromboembolic pulmonary hypertension (CTEPH) is a type of pulmonary hypertension, which is the fourth type, caused by thrombus blocking the pulmonary vascular bed. This leads to pulmonary vascular remodeling and increased pulmonary vascular resistance, resulting in pulmonary hypertension that cannot be resolved [1]. In recent years, Balloon pulmonary angioplasty (BPA) has become a treatment option for CTEPH patients who cannot undergo Pulmonary Endarterectomy (PEA) or who have residual or recurrent pulmonary hypertension after PEA. This method has been shown to improve activity tolerance and hemodynamics in CTEPH patients [2–5]. However, there is limited research on cardiac electrophysiological changes in CTEPH patients following BPA treatment. Therefore, this study aims to analyze the effect of BPA on ECG in CTEPH patients and explore the relationship between ECG and hemodynamics.

## Materials and methods

The retrospective analysis included 39 patients (13 females), mean 61 ± 11 years of age, and body mass index (BMI) mean 23.94 ± 3.37 kg/m^2^. 11 of them received the PAH-specific pharmacotherapy. The inclusion criteria for this study comprised CTEPH patients who had undergone two or more BPA treatments and had available pre- and post-procedure ECG results. The treatment endpoint was defined as the reduction of mean pulmonary artery pressure to normal levels (< 20 mmHg). The standard diagnostic procedures at baseline hospitalization were as follows: physical examination, ECG, laboratory parameters, 6-minute walk distance (6-MWD) and right heart catheterization (RHC). The baseline characteristics of enrolled patients included in our study in Table 1.

**Table 1:** Baseline characteristics of enrolled patients included in the study

Characteristic(*N* = 39)	Patients
Age, years	61 ± 11
Female, n (%)	10 (28.57)
BMI, kg/m2	23.94 ± 3.37
**WHO-FC**,** n (%)**	
Class I	0 (0.00)
Class II	19(48.71)
Class III	19 (48.71)
Class IV	1 (2.56)
pro-BNP, pg/mL	1160.11 ± 2355.94
6MWD (m)	371 ± 92
**Targeted drugs (N = 11%)**	
Phosphodiesterase Type 5 Inhibitor (PDE-5)	1(9.09)
Endothelin receptor antagonist (ERA)	0(0.00)
Soluble Guanylate Cyclase Stimulators (sGCs)	3(27.27)
Soluble Guanylate Cyclase Stimulators (PGI2)	1(9.09)
sGCs + ERA	6(54.55)
ERA + PDE-5	1(9.09)
SGCs + PGI2	1(9.09)
ERA + PDE-5 + PGI2	1(9.09)

Our study compared changes in cardiac function, hemodynamic and ECG related indexes before and after BPA in patients diagnosed with CTEPH and right ventricular hypertrophy. Additionally, the correlation between ECG indexes and hemodynamics was analyzed, specifically screening for ECG indexes significantly correlated with hemodynamic indexes such as mPAP and pulmonary vascular resistance (PVR). The study also assessed the predictive value of these indexes in mPAP greater than or equal to 35 mmHg using ROC curve analysis, and calculated their sensitivity and specificity.Moreover, the CTEPH patients included in this study were segmented into two distinct groups based on the presence or absence of a qR pattern in V_1_. The comparison between the pulmonary hypertension severity-related markers and ECG variables of those with a qR in V_1_ versus those without was subsequently conducted. In addition, for CTEPH patients whose V_1_ lead exhibited qR pattern and their QRS complex electrical axis fell outside the normal range (between − 30 ^°^to + 90^°^), a further division into positive and negative groups was implemented based on whether their electrical axis had returned to within the normal axis range following BPA.

### Electrocardiography

The standard 12-lead ECG was performed at rest on a paper speed of 25 mm/s and a sensitivity of 1 mV = 10 mm. The following parameters were analyzed: heart rhythm, rate, heart axis, intraventricular conduction abnormalities, right bundle branch block (RBBB), RA hypertrophy parameter: qR pattern in V_1_, (R in I + S in III) - (S in I + R in III), highest R in V_1_ or V_2_ + deepest S in I or aVL - S in V_1_, R in V_1_ + deepest S in V_5_ or V_6_, R/S in V_1_, R/S in V_5_. The Butler-Leggett score is calculated by adding the highest R wave in V1 or V2 to the deepest S wave in lead I or aVL, and then subtracting the S wave [6]. The Lewis criteria are defined as (R wave in lead I + S wave in lead III) - (S wave in lead I + R wave in lead III) < 15 mm [7].

### QRS axis

To determine the QRS axis degree on an ECG, first identify the lead with the most isoelectric QRS complex (where the positive and negative deflections are balanced). Next, find the lead perpendicular to it and determine which perpendicular lead shows the most positive deflection. Use the hexaxial reference system to identify the angle corresponding to this positive lead, which gives the QRS axis degree. The normal range of -30° to + 90° reflects the predominant ventricular electrical activity direction.

### qR pattern in lead V_1_

**Fig. 1:**
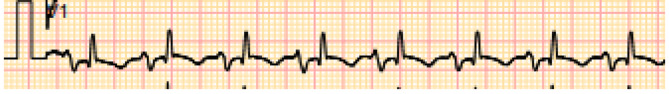
qR pattern in lead V_1_

In lead V1 of the ECG, a qR pattern typically consists of a small, narrow q wave followed by a larger R wave (Fig. 1). The q wave generally has an amplitude of less than 2 mm and a duration of less than 0.04 s, representing the initial depolarization of the interventricular septum. The R wave, which reflects the depolarization of the right ventricle, usually has an amplitude of less than 7 mm.

### Right heart catheterization

The test assessing hemodynamic parameters of the heart was performed with local anesthesia. A Swan-Gantz catheter was inserted through the internal jugular vein into the right heart cavities to assess the following parameters: systolic, diastolic, and mPAP values as well as pulmonary capillary wedge pressure (PCWP) and subsequently to estimate PVR, cardiac output (CO), and cardiac index (CI) .

### Statistical analysis

The statistical analysis for this study was performed using SPSS 23.0. Normally distributed variables were reported as X ± S, while non-normally distributed variables were presented as the median. Categorical variables were expressed as percentages. For comparing means between two groups, paired t-tests or Wilcoxon rank-sum tests were used. Chi-squared tests and composition ratio were used to compare categorical variables, while Mann-Whitney U tests were employed for ordinal categorical variable comparison. Correlation analysis was carried out using Pearson correlation coefficient (R). To evaluate the predictive value of ECG indicators for mPAP, ROC curve analysis was performed.

## Results

### Effects of BPA on Cardiac function indexes and hemodynamics in patients with CTEPH

After BPA, CTEPH patients displayed significant improvements in various areas such as the 6MWD, Pro-BNP levels, and World Health Organization functional class (WHO FC). Additionally, hemodynamically, the treatment resulted in the significant reduction of mPAP, PVR, and total peripheral resistance (TPR). However, the CI and CO did not show any significant improvement after BPA. Table 2 shows a comparison of cardiac function and hemodynamic parameters in all CTEPH patients before and after BPA.

**Table 2:** RHC parameters and cardiac function index before vs. after (*N* = 39)

Variables	Before	After	*P* value
**Cardiac function index**			
WHO FC, (I + II/III + IV)	19/20	32/7	0.092^a^
6MWD, mm	357 ± 88	413 ± 84	0.001
Pro-BNP, pg/ml	1183.62(948.03,1441.93)	441.04(348.62,533.46)	0.024^b^
**RHC**			
SVC (mmHg)	6.23 ± 4.47	5.59 ± 2.36	0.514
mRAP (mmHg)	6.58 ± 5.14	5.64 ± 2.71	0.254
sPAP (mmHg)	70.21 ± 30.26	57.94 ± 22.80	0.000
dPAP (mmHg)	22.65 ± 9.62	18.74 ± 7.77	0.001
mPAP (mmHg)	39.41 ± 15.50	33.24 ± 12.05	0.000
PVR (Wood units)	8.12 ± 5.53	5.61 ± 4.02	0.003
SVR (Wood units)	19.00 ± 7.78	17.85 ± 5.59	0.383
TPR (Wood units)	9.80 ± 5.84	7.26 ± 4.17	0.003
CI ((L/min/m2)	3.10 ± 1.21	3.09 ± 0.70	0.950
CO(L/min)	4,94 ± 2.00	4.99 ± 1.17	0.859

Note: a is the McNemar test, b is the Wilcoxen symbolic rank test, and the other is the paired T test

### Effect of BPA on Electrocardiogram in patients with CTEPH

After BPA, The number of CTEPH patients with qR pattern in V_1_ and deep S in V_6_ were significantly reduced. The number of CTEPH patients with QRS axis deviation also decreased significantly .However, there were no significant changes in other indicators such as heart rate, P in II, S in V_5_, Butler-Leggett score, and Lewis criteria after BPA. A comparison of all specific ECG parameters is provided in Table 3.

**Table 3:** ECG parameters before vs. after (*N* = 39)

Variables	Before	After	*P* value
HR(times)	77 ± 14	73 ± 12	0.056*
QRS axis (°)	100(90,106)	96(88,110)	0.001
The duration of QRS complex (ms)	100(90,106)	96(88,110)	0.992
P in II (µV)	130(108,170)	123(90,145)	0.503
R in V_1_ (µV)	400(150,620)	270(150,640)	0.461
R in V_5_ (µV)	1150(740,1630)	1160(820,1710)	0.105
S in V_5_ (µV)	750(390,1110)	620.00(400,1070)	0.222
S in V_6_ (µV)	440(220,760)	350(220,670)	0.042
**AHA/ACCF/HRS Criteria for Right Ventricular Hypertrophy Electrocardiogram Diagnosis**			
qR in V1	12(30.77)	3(7.69)	0.014
R in V_1_ > 6 mm, n(%)	12(30.77)	10(25.64)	1.000
R/S in V_1_ > 1, n(%)	15(38.46)	11(28.21)	0.739
S in V_5_ > 10 mm, n(%)	12(30.77)	11(28.21)	0.763
R in aVR > 4 mm, n(%)	6(15.38)	1(2.56)	0.102
S in V_1_ < 2 mm, n(%)	10(25.64)	10(25.64)	0.739
R/S V_5_ < 0.75, n(%)	4(10.25)	2(5.12)	0.414
R/S in V_5_ / R/S in V_1_ < 0.04, n(%)	1(2.56)	0(0.00)	0.317
Lewis criteria	39(100.00)	39(100.00)	1.000
Butler-Leggett score	25(64.11)	23(58.97)	0.705

Note: * is paired T test, others are Wilcoxen symbolic rank test

### Correlation between Electrocardiographic Parameters and hemodynamic parameters

**Fig. 2:**
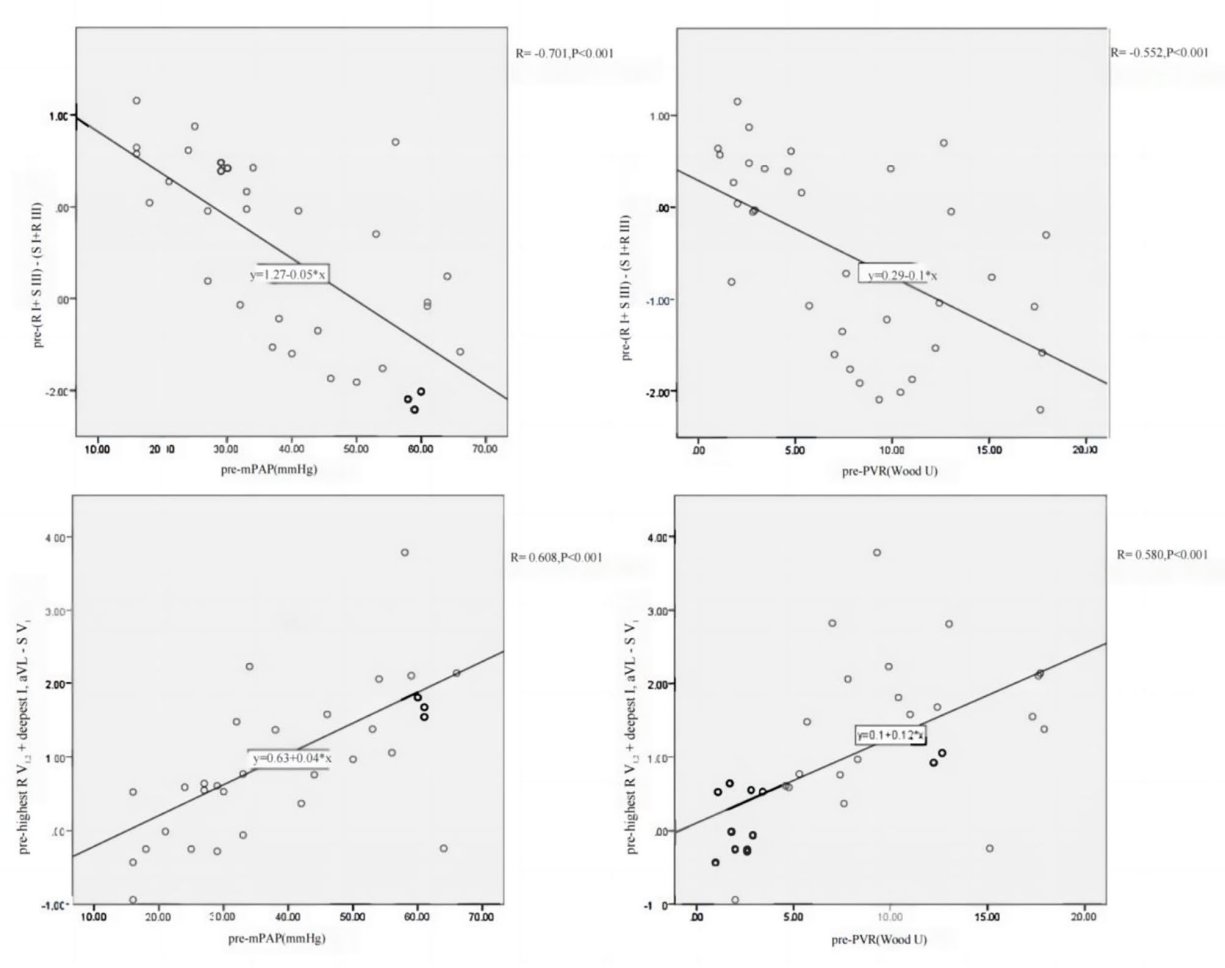
Correlation between ECG parameters and hemodynamics

**Fig. 3:**
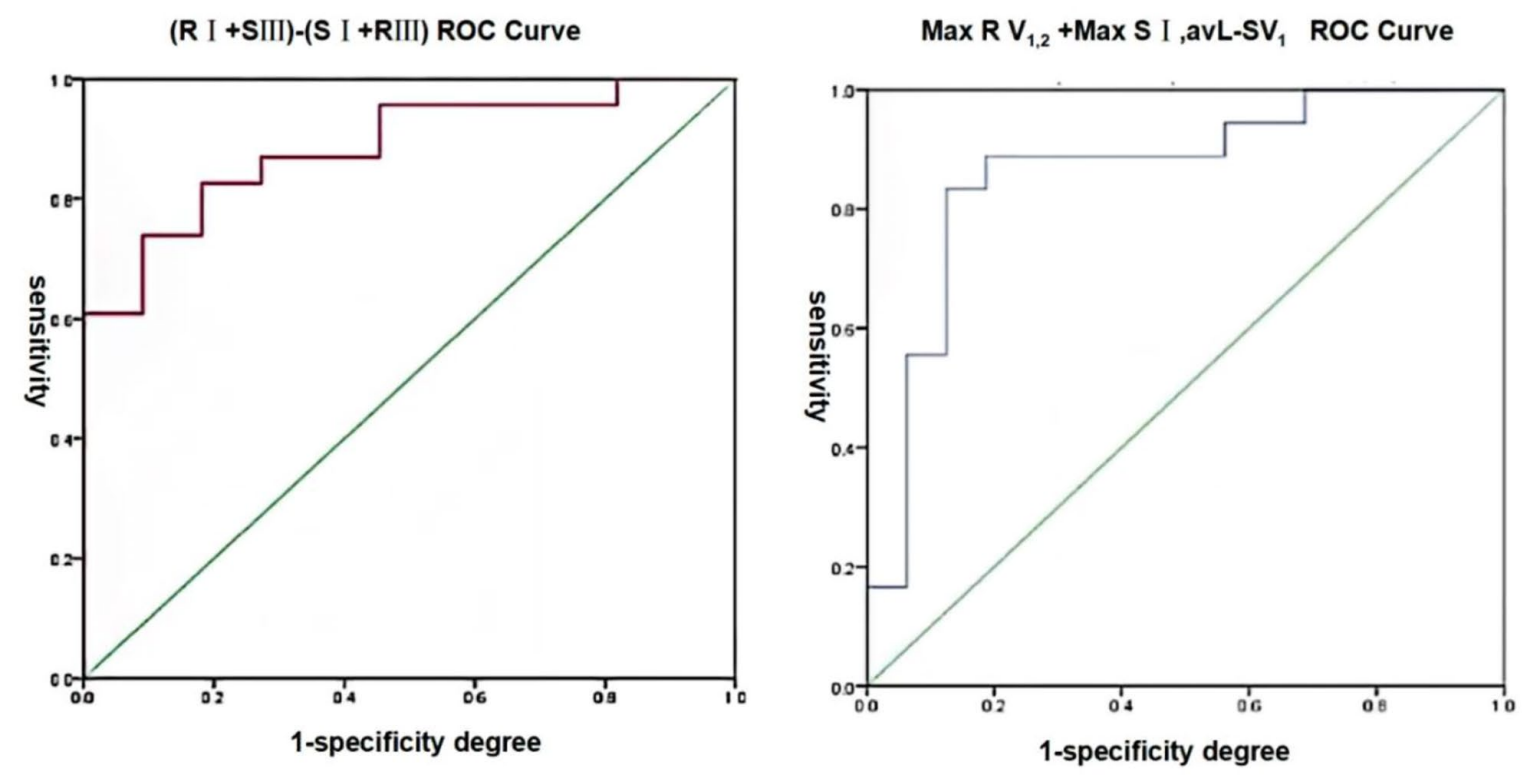
ROC Curve of Electrocardiogram Index versus mPAP ≥ 35mmHg.

A significant correlation was observed between hemodynamics and specific electrocardiographic (ECG) parameters associated with right ventricular hypertrophy, namely qR pattern in V_1_, Lewis criteria, and Butler-Leggett score (Fig. 2). To assess the predictive capability of the Lewis criteria and the Butler-Leggett score for mPAP ≥ 35mmHg, ROC curve analysis was employed. The findings revealed that both the Lewis criteria and the Butler-Leggett score exhibited strong predictive potential, with area under the curve values of 0.885 and 0.861, respectively, for mPAP ≥ 35mmHg (Fig. 3).

### Change of QRS Axis in patients with qR pattern in V_1_

A total of 25 cases with QRS axis deviation beyond the normal range were identified among CTEPH patients. Following BPA, complete normalization of QRS axis (within the range of -30° to + 90°) was observed in 14 cases (56%). Among the 12 patients with qR pattern in V_1_, BPA resulted in positive outcomes (conversion to a normal QRS axis) in 9 cases (75%), while 3 cases (25%) remained negative (QRS axis did not normalize). Notably, in the positive cases, the disappearance of the qR pattern in V_1_ was observed in 9 cases (75%), accompanied by a restoration of the abnormal axis to the normal range. Cardiac ultrasound revealed a regression in the size of the right atrium and right ventricle, indicating a return to normal dimensions.

### **The Relationship between qR Pattern in V**_**1**_**and Pulmonary arterial hypertension severity and Electrocardiographic Variables**

The study involved dividing the CTEPH patients into two groups based on the presence or absence of a qR wave pattern in V_1_. The severity of pulmonary arterial hypertension (PAH) and electrocardiographic variables were compared between the qR V_1_ and no-qR V_1_ groups. The findings demonstrated a significant increase in mean pulmonary artery pressure (mPAP) and pulmonary vascular resistance (PVR) in the qR V_1_ group compared to the no-qR V_1_ group. Additionally, cardiac index (CI) and cardiac output (CO) were found to be significantly lower in the qR V_1_ group compared to the no-qR V1 group. Regarding electrocardiographic parameters, there were statistically significant differences in Butler-Leggett score and R in aVR between the two groups. Specifically, the qR V_1_ group exhibited a significantly higher BL score compared to the no-qR V_1_ group. Table 4 provides a comparison of the two specific parameters.

**Table 4:** qR V1 Group vs. no-qR V1 Group

Variables	qR V_1_	no-qR V_1_	*P* value
N,%	12(25.71)	27(74.29)	
Age, years	58 ± 11	62 ± 11	0.409
**Cardiac function index**			
WHO FC, (I + II/III + IV)	4/8	16/11	0.262^a^
6MWD, m	354 ± 78	377 ± 98	0.620
Pro-BNP, pg/ml	2913.41(2154.75,3672.07)	546.00(308.00,784.00)	0.089^b^
**RHC**			
mRAP (mmHg)	9.88 ± 8.81	5.52 ± 2.79	0.208
mPAP (mmHg)	51.56 ± 11.13	35.04 ± 14.63	0.004
PVR (Wood units)	12.70 ± 4.05	6.16 ± 4.70	0.001
CI ((L/min/m2)	2.22 ± 0.65	3.42 ± 1.22	0.009
CO(L/min)	3.50 ± 0.92	5.40 ± 2.06	0.001
**ECG parameters**			
HR(times)	85 ± 12	75 ± 14	0.208
QRS axis (°)	139 ± 54	70 ± 63	0.006
P in II (mV)	0.17 ± 0.04	0.12 ± 0.08	0.061
R in aVR (mV)	0.28 ± 0.15	0.15 ± 0.15	0.029
Lewis criteria	-1.10 ± 0.87	-0.39 ± 1.02	0.071
Butler-Leggett score	1.84 ± 0.67	0.76 ± 1.03	0.006
R in V_1_ + deepest S V_5_ or V_6_ (mV)	1.63 ± 1.10	1.08 ± 0.66	0.085

Note: a is the McNemar test, b is the Wilcoxen symbolic rank test, others are independent sample T test

## Discussion

After more than three decades of technical improvements and clinical practice, the efficacy and safety of BPA has been confirmed to some extent, especially in its ability to improve hemodynamics and exercise tolerance (8–9). In our study, hemodynamics and activity tolerance of CTEPH patients were significantly improved after BPA. Although 62.86% of the patients in this study did not reach the treatment endpoint, parameters such as 6MWD, WHO FC, and Pro-BNP were significantly improved in these patients. In addition, CTEPH patients can experience reversal of myocardial remodeling and reduced dyssynchrony, which may improve right ventricular function to some extent after undergoing balloon pulmonary angioplasty (BPA) treatment [9–11]. Furthermore, it has even been shown that the right heart function may still can be improved in CTEPH patients who undergo BPA even if the mPAP is normalized [12]. Additionally, the relatively low mean right atrial pressure in this study’s patients suggests that most had preserved right ventricular function prior to the intervention. Consequently, there was no significant decrease in mean right atrial pressure post-intervention (*P* > 0.05). However, a downward trend in mean right atrial pressure was still observed after BPA treatment, suggesting that BPA may partially improve right ventricular function in patients with CTEPH.

ECG can reflect right ventricular hypertrophy and serves as a tool to evaluate myocardial remodeling reversal. Additionally, it is an independent predictor beyond hemodynamic parameters and cardiac function classification [13]. In CTEPH, which belong to Type 4 of pulmonary hypertension types, increased pulmonary vascular resistance causes right heart remodeling, resulting in corresponding changes in the ECG reading. (R in I + S in III) - (S in I + R in III) < 15 mm is also known as the Lewis criteria [6]. In the MESA-right ventricular study, Whitman indicated that electrocardiographic criteria for patients with right ventricular hypertrophy have higher specificity but lower sensitivity. The Lewis criteria had the highest sensitivity (80.4%) among 22 indicators, but their specificity was the lowest (16.8%). However, since it included patients with mitral stenosis and aortic valve disease, the Lewis criteria are not easily applicable to the general population. Furthermore, the positive and negative predictive values of the Lewis criteria were poor in the MESE - right ventricular study [14]. However, it should be noted that the incidence of positive Lewis criteria was 100% in our study population and was significantly associated with mPAP and PVR. The Lewis criteria demonstrated good predictive value for predicting mPAP ≥ 35mmHg, with high sensitivity and specificity. Furthermore, the study population specifically consisted of patients with known pulmonary vascular disease. As such, the Lewis criteria can accurately reflect the hemodynamic severity of patients with CTEPH, and since it is derived from limb leads, it can prevent data errors resulting from inconsistent lead placement, enabling more accurate and repeatable ECG diagnoses and follow-up of patients with right ventricular hypertrophy. Highest R in V_1_ or V_2_ + deepest S in I or aVL - S in V_1_ is another electrocardiographic index with significant correlation with hemodynamics in addition to Lewis criteria, which is called Butler-Leggett score [7]. Blyth et al. conducted a study comparing cardiac magnetic resonance imaging and electrocardiography in 28 patients with pulmonary hypertension, and concluded that the Butler-Leggett score is a highly specific yet insensitive index for detecting right ventricular hypertrophy [15]. In our study, the positive rate of Butler-Leggett score in patients with CTEPH included was 62.86%, which was significantly correlated with mPAP and PVR, and had good clinical value for predicting mPAP ≥ 35mmHg. However, due to the lack of cardiac magnetic resonance imaging concerning right ventricular hypertrophy, it was not possible to evaluate the relationship between Butler-Leggett score and right ventricular hypertrophy.

Apart from the evaluation of right ventricular remodeling, there is still limited research on whether electrocardiography can assess the therapeutic effect of BPA in CTEPH patients. Ghio et al. compared the right ventricular hypertrophy-related indices of electrocardiography before and after PEA in 99 CTEPH patients, and found that they were consistent with the rapid improvement of hemodynamics and persistent reversal of myocardial remodeling reflected by echocardiography [16]. Unlike PEA, BPA involves staged balloon dilation of narrowed pulmonary arteries. Hemodynamic improvement in CTEPH patients with BPA occurs gradually over multiple procedures.

Fukui et al. found the reversal of right ventricular remodeling in CTEPH patients 3–6 months after the last BPA procedure through comparison of cardiac magnetic resonance imaging before and after BPA [17]. Moreover, Tokgöz HC et al. found that the P wave amplitude, R wave amplitude in aVR, right or indeterminate axis deviations, and R/S ratio in V1 and V2 showed statistically significant correlations with pulmonary hemodynamics [18]. Our study identified a significant reduction in the number of patients with qR pattern in V_1_ or deep S pattern in V_6_ among CTEPH patients after BPA. The observed decrease in such patients following BPA demonstrated statistical significance.The mechanism underlying the presence of qR pattern in V_1_ among pulmonary arterial hypertension (PAH) patients remains unclear. We speculate that the appearance of qR pattern in V_1_ lead may be due to significant clockwise rotation of the heart resulting from severe right ventricular hypertrophy. Although the degree of right ventricular hypertrophy was not measured by cardiac magnetic resonance imaging in this study, patients in the qR V_1_ group had higher Butler-Leggett scores, which are believed to be related to right ventricular mass [15]. Therefore, it is possible that qR pattern in V_1_ lead is associated with right ventricular hypertrophy.

In our study, significant correlations were found between qR pattern in V_1_ and mPAP, PVR. Patients in the qR V_1_ group had higher mPAP and PVR compared to those in the no-qR V_1_ group, with significantly decreased CO and CI. It was concluded that CTEPH patients with a qR pattern in V_1_ have worse hemodynamic status compared to those without qR pattern in V_1_, with increased PVR and right ventricular pressure overload, leading to poor prognosis. Nagai et al. found a significant correlation between qR pattern in V_1_ and the right ventricular/left ventricular volume ratio, as well as the diastolic eccentricity index. The probability of having right ventricular systolic dysfunction was higher in pulmonary arterial hypertension patients with a qR pattern in V_1_ compared to those with normal right ventricular systolic function, making it an important predictor for right ventricular systolic dysfunction [19]. Some studies have suggested that qR pattern in V_1_ may be a sign of advanced pulmonary arterial hypertension, significantly correlated with the patient’s cardiac function status, hemodynamics and severity of right heart dysfunction. The Cox regression analysis showed that, apart from PVR and CI, qR pattern in V1 could also serve as a predictor for mortality risk in this population [20, 21]. In our study, among all the AHA/ACCF/HRS electrocardiographic criteria for right ventricular hypertrophy (RVH), the number of CTEPH patients who presented qR pattern in V_1_ and S pattern in V_6_ with amplitudes greater than 3 mm showed a significant reduction after BPA. However, previous studies have found that P in II, S in V_5_, and R in V_5_ of CTEPH patients also exhibited significant reduction in amplitude after BPA [22–25]. Unlike previous studies, it should be noted that the patients included in our study underwent fewer BPA procedures. In CTEPH patients who did not achieve their treatment goals, the qR pattern in V_1_ disappeared from electrocardiogram, suggesting that the disappearance of this ECG indicator may occur earlier during BPA in CTEPH patients. Furthermore, similar to the qR pattern in V_1_, the QRS axis deviation in CTEPH patients could also return to the normal range at an early stage. The normal range of -30° to + 90° reflects the predominant ventricular electrical activity direction. The return of the QRS axis degree to the normal range (-30° to + 90°) indicates that the ventricular function of CTEPH patients has improved or recovered following BPA treatment. We believe it may be associated with the improvement of right ventricular hypertrophy in patients after BPA. Therefore, this study indicates the early reversal of QRS axis deviation and the early disappearance of the QR pattern in lead V1 may serve as valuable indicators for assessing post-BPA satisfaction in CTEPH patients. Both are valuable for evaluating and predicting hemodynamic improvement following BPA.

## Data Availability

No datasets were generated or analysed during the current study.
